# A Window Opens and a Shunt Closes: A New Laparoscopic Approach for the Attenuation of the Gastrophrenic Shunt

**DOI:** 10.3390/vetsci12040351

**Published:** 2025-04-09

**Authors:** Brenda Viviane Götz Socolhoski, Amanda Oliveira Paraguassú, Franciéli Mallmann Pozzobon, Pâmela Caye, Jean Carlos Gasparotto, Otávio Henrique de Melo Schiefler, Rainer da Silva Reinstein, Daniel Curvello de Mendonça Müller, Maurício Veloso Brun

**Affiliations:** Postgraduate Program in Veterinary Medicine, Center for Rural Sciences, Federal University of Santa Maria (UFSM), Santa Maria 97105-970, Brazil; amanda.medicinavet@gmail.com (A.O.P.); f.ramp@hotmail.com (F.M.P.); pamiscaye@gmail.com (P.C.); jeangasparotto@hotmail.com (J.C.G.); vetotavio@gmail.com (O.H.d.M.S.); rainerreinstein@gmail.com (R.d.S.R.); mullerdcm@gmail.com (D.C.d.M.M.); mauriciovelosobrun@hotmail.com (M.V.B.)

**Keywords:** ameroid constrictor, dogs, portosystemic shunt, hepatopathy, intracorporal suture, video surgery

## Abstract

The portosystemic shunt is characterized by the presence of an anomalous vessel, whether congenital or acquired, that connects the portal circulation to the systemic circulation. Surgical treatment is indicated in congenital cases and involves the progressive occlusion of the anomalous vessel. The objective of this study is to report the use of an innovative and fully laparoscopic technique for the attenuation of a gastrophrenic shunt. The procedure was performed on a two-year-old female German Spitz dog with a history of frequent vomiting and diarrhea. The diagnosis was established through computed tomography. For the procedure, three portals were placed in the left lateral region, employing an ameroid constrictor. This device featured a specific perforation for the passage of a pre-mounted suture, which was subsequently occluded intracorporeally, facilitating the closure of the ring defect. The absence of flow in the anomalous vessel was confirmed five weeks after the procedure. It is concluded that the reported implantation technique represents a new modality for the treatment of this type of shunt using a minimally invasive approach.

## Simple Summary

A portosystemic shunt is an anomalous vessel that connects the portal circulation to the systemic circulation, reducing the flow of blood drained from the gastrointestinal system to the liver and generating significant clinical implications. Treatment consists of gradual occlusion of the shunt, aiming at the restoration of liver function and resolution of the clinical signs presented. The ameroid constrictor (AC) is widely used for this purpose; however, to date, there are no reports of its laparoscopic application. This study demonstrates the feasibility of the totally laparoscopic application of an AC associated with intracorporeal sutures in a canine affected by a gastrophrenic shunt.

## 1. Introduction

The deviation or portosystemic shunt is characterized by the presence of an anomalous vessel, congenital or acquired, communicating the portal circulation to the systemic [1,2]. This condition reduces the blood flow drained from the gastrointestinal system to the liver, decreasing the hepatotrophic factors and allowing the toxin passage to the systemic circulation, such as ammonia, aromatic amino acids (phenylalanine, tyrosine, and tryptophan), short-chain fatty acids, mercaptans and various biogenic amines, indoles, and skatoles [3], leading to significant clinical implications, such as hepatic encephalopathy or hepatic insufficiency [2,4,5]. The reduction of liver oxygenation due to blood reduction through the portal vein is also associated with the condition [2,6].

Based on their anatomical location, portosystemic shunts are classified as intrahepatic or extrahepatic [2,7,8,9]. Their definition is according the portal vessel from which they originate and the systemic vein into which they insert [7]. The evaluation of the portal system can be performed using ultrasonography, splenoportography, computed tomography, or magnetic resonance angiography [10].

Surgical treatment of portosystemic shunts is indicated in congenital cases and primarily consists of occluding the anomalous vessel to restore hepatic function and resolve the associated clinical signs [1,2]. Gradual attenuation using an ameroid constrictor (AC) has been widely employed in dogs [11,12], as it facilitates hepatic adaptation to increased blood flow, thereby preventing the development of acute portal hypertension [1,13].

Minimally invasive approaches for the treatment of extrahepatic shunts have been reported, contributing to reduced morbidity [14,15,16]. However, to the authors’ knowledge, there are no previous reports on the application of an AC via a fully laparoscopic approach for the attenuation of phrenic shunts in dogs. In this context, the present study aims to describe an innovative and effective purely laparoscopic approach for the attenuation of a gastrophrenic shunt in a female German Spitz dog.

## 2. Materials and Methods

A two-year-old female German Spitz dog, intact and weighing 2.1 kg, presented with a history of frequent vomiting and diarrhea. According to the owners, the patient also exhibited poor physical development relative to its age group. Based on the clinical history, an ultrasonographic evaluation was performed, revealing a slight reduction in hepatic volume. Additionally, hematological analysis showed evaluated serum levels of alanine aminotransferase (341 U/L), alkaline phosphatase (989 U/L), ureia (21 mg/dL), and creatinina (1.1 mg/dL).

**Figure 1:**
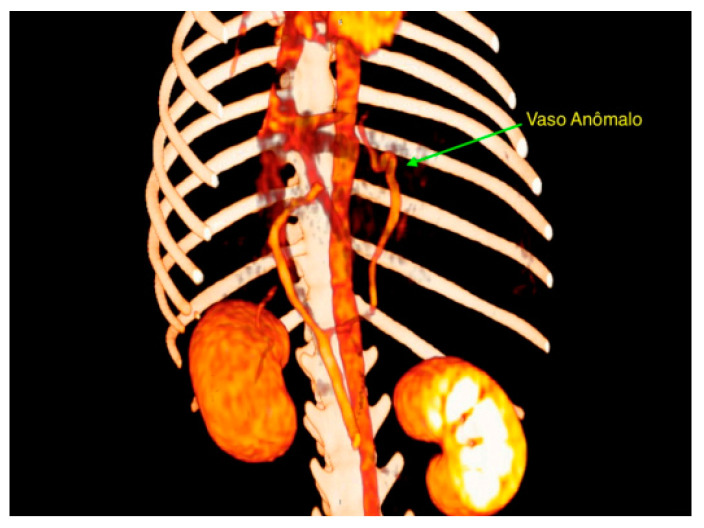
Tomographic images of the anomalous vessel (arrow) originating from the left gastric vein.

Given the clinical history and observed signs, a contrast-enhanced computed tomography was performed, identifying an anomalous vessel originating from the left gastric vein, coursing cranially adjacent to the gastric fundus, and inserting into the phrenic vein at the level of the diaphragm (Figure 1). Based on these findings, medical management was initiated, including lactulose therapy, a gastrointestinal diet, and a recommendation for surgical correction.

For the procedure, the patient was maintained under general inhalation anesthesia and positioned in dorsal recumbency with a rightward tilt. The first trocar (11 mm) was inserted in the left paramedian region using the open technique (modified Hasson), followed by insufflation of the abdominal cavity with carbon dioxide and a pressure of 6 mmHg. A 10 mm, 30-degree rigid endoscope was used. The second and third trocars, both 6 mm, were placed in a triangulated arrangement on the left abdominal wall, directed cranially.

**Figure 2:**
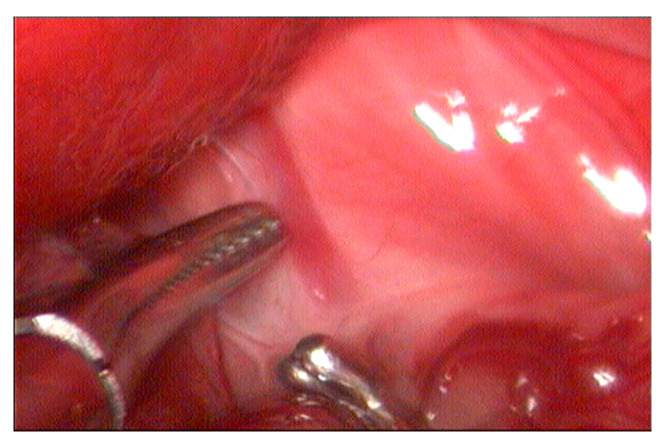
The shunt (the tip of the forceps) visualized near the diaphragm.

Microhepatia and irregular hepatic lobes were observed, along with the anomalous vessel near the diaphragm (Figure 2). Careful dissection of the vessel was performed using a 5 mm laparoscopic Kelly forceps and a right-angled Mixter forceps. Following its isolation, a 3.5 mm AC with dual perforations in the casein and without its locking pin and a pre-mounted polypropylene 2-0 suture loop positioned in one of the ring’s openings was introduced through the 11 mm port, which was temporarily disassembled. The cavity was subsequently re-insufflated at the previously described pressure.

**Figure 3:**
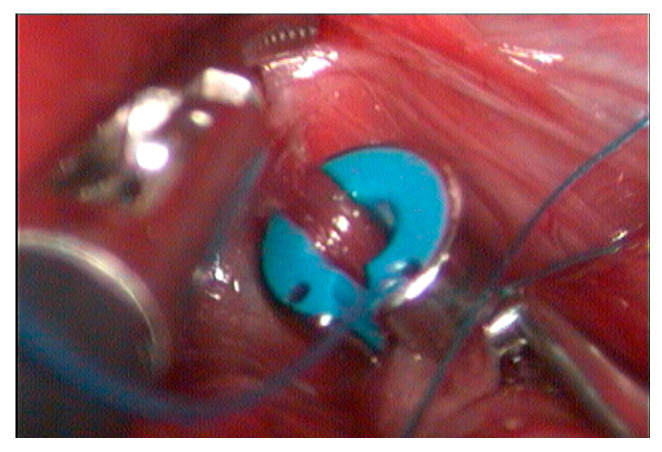
The ameroid ring encircling the shunt near its passage through the diaphragm.

**Figure 4:**
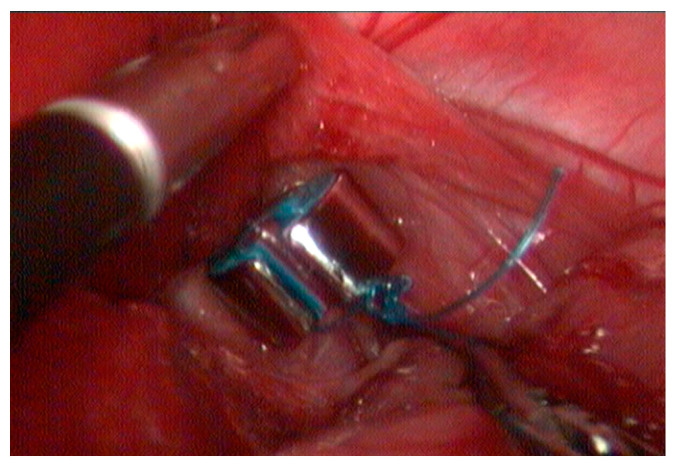
The final aspect of the ameroid ring closure with an intracorporeal knot.

The device was implanted, encircling the shunt near its passage through the diaphragm (Figure 3). The pre-formed suture loop was then sectioned. Subsequently, the free end of the suture was introduced into the other opening of the ring. Using a needle holder and counter-needle holder, intracorporeal occlusion suturing was carried out (Figure 4) with five half-knots. Four hepatic biopsies were also performed. The total duration of the procedure was one hour and eight minutes from the incision of the first portal to the completion of the cavity closure.

Postoperative care included the administration of dipyrone (25 mg/kg, TID, PO, for three days), tramadol hydrochloride (2 mg/kg, TID, PO, for two days), and meloxicam (0.1 mg/kg, SID, PO, for two days). Additional recommendations included rest, the use of surgical vest, and wound cleaning with 0.9% NaCl solution until suture removal.

## 3. Results

The patient was discharged on the same day without exhibiting signs consistent with portal hypertension during the intraoperative or postoperative periods. After five weeks, a follow-up ultrasonographic exam confirmed the absence of blood flow within the anomalous vessel, as reported via telephone contact.

## 4. Discussion

The portal vascular anatomy in dogs and cats with congenital extrahepatic shunts is generally considered normal except for the anomalous connection between the portal and systemic venous systems [17]. To accurately characterize portal vasculature anatomy, several studies have concluded that in the most common types of congenital shunts, the involved veins are normal vessels within the portal venous system [17,18,19]. In the present case, the patient exhibited an anomalous vessel originating from the left gastric vein, inserting into the phrenic vein, which subsequently drained into the caudal vena cava, thereby characterizing a left gastrophrenic shunt.

In a study by White and Parry [19], gastrophrenic shunting accounted for 61% of congenital shunts originating from the left gastric vein in dogs and cats. In broader studies on extrahepatic shunting in dogs, White et al. [17] reported an incidence of 46%. Within this context, the present case appears atypical. In the authors’ clinical experience, the majority of cases involve shunting to the abdominal caudal vena cava rather than to the phrenic or azygos veins.

Both the clinical signs and observed laboratory abnormalities are nonspecific and highly variable, influenced by the type of shunt, vascular anatomy, nutritional status, and concomitant diseases [20,21]. These alterations occur due to a significant portion of portal blood being diverted away from the liver, leading to a reduced supply of hepatotrophic factors and, consequently, decreased hepatic volume and compromised metabolic function [2,6]. This phenomenon may explain the elevated hepatic enzyme levels and the presence of periportal fibrosis, suggestive of chronic hepatopathy and moderate biliary hyperplasia, as observed in the histopathological evaluation.

Clinical signs typically manifest around two years of age, which corresponds with the age of the patient in this case, and are primarily associated with the gastrointestinal and nervous systems [13]. Affected animals may present with diarrhea, emesis, polyuria, polydipsia, and hematuria due to the formation of ammonium biurate crystals. Additionally, neurological signs related to hepatic encephalopathy such as compulsive walking, head pressing, ataxia, lethargy, and stupor, may be observed. Growth retardation is also expected [13,18]. In the present case, the patient exhibited gastrointestinal symptoms, including frequent episodes of vomiting and diarrhea and the appearance of underdevelopment to that point, which prompted the owners to seek veterinary care.

The patient in question did not present neurological signs. However, according to Gow [3], such signs are commonly present; however, if of low intensity, they may be subtle and expressed only through mild and nonspecific cortical inhibition or changes in behavior, making it difficult to assess the neurological impact of this vascular anomaly. In dogs, a congenital portosystemic shunt has been associated with marked cellular changes in the cerebral cortex and cerebellum [22]. In this context, a case study carried out by Stefănescu et al. [5] demonstrated that electroencephalography (EEG) can complement the diagnosis of portosystemic shunts and may be useful in the therapeutic protocol and in the assessment of changes in cerebral electrical homeostasis. However, such an examination was not performed.

Clinical management can control symptoms; however, it does not completely eliminate them, and therefore, surgical treatment is associated with increased patient survival and is recommended in most cases [23,24]. Clinical strategies include the administration of non-absorbable disaccharides (lactulose), antibiotics, and dietary modifications, which are primarily indicated for pre-surgical stabilization or when surgical intervention is not feasible [21,25]. Lactulose enhances intestinal content elimination and acidifies the lumen, facilitating the conversion of ammonia into ammonium, thereby reducing its systemic levels and preventing the development of hepatic encephalopathy [25].

Surgical techniques have been described for the occlusion of extrahepatic portosystemic shunts, including ligation with suture material, the use of an AC, cellophane bands, hydraulic occluders, and intravascular approaches [11,12]. Nevertheless, ameroid rings and cellophane bands remain the most commonly used and reported methods in dogs, demonstrating satisfactory outcomes [26]. To the best of the authors’ knowledge, this report is the first purely laparoscopic approach using CA. However, a study by Poggi et al. [15] evaluated complications and the outcome of total laparoscopic portosystemic shunt attenuation using a polymer-coated cellophane band or a thin film strip from commercially available cigarette packs sterilized with ethylene oxide. In conclusion, the authors mentioned that the technique would be feasible in dogs, especially for shunts located in the epiploic foramen. However, portal hypertension was observed in four of the twenty patients evaluated and conversion to celiotomy was necessary in one-third of the cases.

Regarding its structural characteristics, the AC consists of a metallic semicircle enclosing a dehydrated casein ring, which gradually expands upon rehydration with bodily fluids [13]. In the present case, the AC used featured dual perforations in the casein component to facilitate the passage of the suture material, which served as a closure mechanism (after performing the half-knots), mimicking the function of the standard locking pin. To the authors’ knowledge, the introduction and locking of the pin is the greatest complicating factor in the laparoscopic attenuation of portosystemic shunts, as it is a small piece and difficult to handle with conventional laparoscopic forceps inside the abdominal cavity. In this context, it is noted that the technique represents a potential alternative to this limitation, enabling the use of the standard CA (slightly modified) of proven efficacy and highlighting the beneficial impact of the minimally invasive approach in the treatment of portosystemic shunts in dogs. The implementation method followed the principles described by Brun e Pasquale [27], representing a fully laparoscopic, innovative technique associated with reduced morbidity and early patient discharge.

This case represents the first report of this novel approach for a shunt involving the phrenic vein. Consequently, the placement of the trocars on the left abdominal side and patient orientation in dorsal recumbency with a slight right tilt differed from previously described occlusion techniques for shunts draining into the abdominal caudal vena cava [16]. According to the authors, the passage of the suture through the perforations and intracorporeal knot placement were facilitated in this approach compared to AC placement for shunts draining into the abdominal caudal vena cava cranial to the phrenicoabdominal vessels [27].

The gradual attenuation of the shunt following AC placement depends on the reaction of adjacent soft tissues [1], particularly the inflammatory response and thrombosis stimulated by the implant [28]. Due to its hygroscopic nature, the rehydration of casein by bodily fluids induces its expansion and the progressive occlusion of the anomalous vessel [13]. While larger-diameter constrictors may fail to achieve complete occlusion, smaller-diameter constrictors carry an increased risk of postoperative portal hypertension [1]. In the present case, the AC was carefully selected based on a detailed assessment of the shunt’s systemic insertion diameter using computed tomography, with the aim of placing the implant as close as possible to the vessel’s passage through the diaphragm.

The ideal timeframe for complete closure of a portosystemic shunt remains uncertain [11], with reported periods ranging from two weeks to three months [13]. In another study, researchers observed a 31% reduction in the AC’s internal diameter within eight weeks postoperatively via computed tomography [26]. Ultrasonography is the primary imaging modality for evaluating portosystemic shunts [4] and can be used to assess postoperative occlusion [29]. However, computed tomography angiography provides high-resolution images, offering a more comprehensive view of portal vascular anomalies and is thus considered superior for this purpose [18]. In the present case, only an ultrasonographic assessment was performed. However, direct visualization for the AC was not possible due to surrounding visceral tissue. This observation underscores the higher diagnostic reliability of computed tomography for postoperative evaluation in similar cases. Nevertheless, the cost–benefit ratio must be carefully considered, as ultrasonography is more cost-effective, does not require general anesthesia, and generally provides sufficient information regarding blood flow—or its absence—through the anomalous vessel.

Regarding the incidence of complications, the persistence of clinical signs due to incomplete occlusion of the anomalous vessel and portal hypertension caused by acute occlusion of the shunt are the most common adverse events, making the prognosis poor [30]. Thus, it is highlighted that progressive closure until complete occlusion of the vessel reduces the risk of complications, making the CA an excellent option. However, according to Park et al. [31], CA migration can occur up to one year after its implantation and its displacement can be attributed to gravitational compression of the shunt, causing abdominal pain and intestinal obstruction. Human studies suggest that the effect of gravity or body movement and the use of relatively large implants can contribute to implant sliding and migration [32,33]. Information on CA displacement in dogs is scarce, highlighting the need for more in-depth investigations and longer and more careful clinical monitoring.

## 5. Conclusions

It is concluded that the technique of implanting the ameroid constrictor via a fully laparoscopic approach is promising in cases of phrenic portosystemic shunts and can be added to the list of minimally invasive therapeutic possibilities for the treatment of this disease in dogs. However, the authors recognize the need for more comprehensive studies to confirm its real effectiveness in dogs.

## Data Availability

Data are contained within the article.
